# Correction to: Predicting Radiotherapy Patient Outcomes with Real-Time Clinical Data Using Mathematical Modelling

**DOI:** 10.1007/s11538-024-01268-2

**Published:** 2024-02-28

**Authors:** Alexander P. Browning, Thomas D. Lewin, Ruth E. Baker, Philip K. Maini, Eduardo G. Moros, Jimmy Caudell, Helen M. Byrne, Heiko Enderling

**Affiliations:** 1 Mathematical Institute, University of Oxford, Oxford, UK; 2 Roche Pharma Research and Early Development, Roche Innovation Center, Basel, Switzerland; 3 Department of Radiation Oncology, H. Lee Moffitt Cancer Center & Research Institute, Tampa, USA; 4 Department of Integrated Mathematical Oncology, H. Lee Moffitt Cancer Center & Research Institute, Tampa, USA; 5 Present Address: Department of Radiation Oncology, MD Anderson Cancer Center, Houston, TX USA

**Correction to: Bulletin of Mathematical Biology (2024) 86:19** 10.1007/s11538-023-01246-0

In this article the author name Jimmy Caudell was incorrectly written Jimmy Chaudell in the original version of this article.

The author group has been corrected above and the original article has been corrected.

