# Supplementary material for: Predicting Radiotherapy Patient Outcomes with Real-Time Clinical Data Using Mathematical Modelling
Source: Bull Math Biol. 2024 Jan 18;86(2):19. doi: 10.1007/s11538-023-01246-0 (PMC10796515; doi:10.1007/s11538-023-01246-0)
Supplement: Supplementary file 1 — Supplementary information This article contains one electronic supplementary material file. (pdf 4,584KB) [file 11538_2023_1246_MOESM1_ESM.pdf]

Supplementary material for "Predicting radiotherapy patient outcomes with real-time clinical data using mathematical modelling"

Alexander P Browning[*‡1], Thomas D Lewin[*1,2], Ruth E Baker[1], Philip K Maini[1], Eduardo G. Moros[3], Jimmy Caudell[3], Helen M Byrne[†1], and Heiko Enderling[†3,4,5]

[1] *Mathematical Institute, University of Oxford, Oxford, UK*
[2] *Roche Pharma Research and Early Development, Roche Innovation Center, Basel, Switzerland*
[3] *Department of Radiation Oncology, H. Lee Moffitt Cancer Center & Research Institute, USA*
[4] *Department of Integrated Mathematical Oncology, H. Lee Moffitt Cancer Center & Research Institute, USA*
[5] *New Address: Department of Radiation Oncology, MD Anderson Cancer Center, Houston, TX, USA*

January 10, 2024

Table S1. Parameters resampled from the full posterior corresponding to four synthetic patients.

Parameter	Fast responder	Poor responder	Plateaued responder	Pseudo progression
λ	$3.5 \times 10^{-2}\,\mathrm{d}^{-1}$	$3.0 \times 10^{-1}\,\mathrm{d}^{-1}$	$3.2 \times 10^{-1}\,\mathrm{d}^{-1}$	$3.2 \times 10^{-1}\,\mathrm{d}^{-1}$
K	2.2	2.2	1.1	1.5
γ	$1.1 \times 10^{-1}\,\mathrm{d}^{-1}$	$1.0 \times 10^{-2}\,\mathrm{d}^{-1}$	$2.2 \times 10^{-1}\,\mathrm{d}^{-1}$	$4.2 \times 10^{-1}\,\mathrm{d}^{-1}$
ζ	$1.4 \times 10^{-1}\,\mathrm{d}^{-1}$	$2.4 \times 10^{-4}\,\mathrm{d}^{-1}$	$3.6\,\mathrm{d}^{-1}$	$4.5 \times 10^{-2}\,\mathrm{d}^{-1}$
η	$1.6 \times 10^{-4}\,\mathrm{d}^{-1}$	$8.3\,\mathrm{d}^{-1}$	$1.3 \times 10^{-4}\,\mathrm{d}^{-1}$	$1.4 \times 10^{-4}\,\mathrm{d}^{-1}$
ϕ_i	9.4×10^{-2}	1.5×10^{-1}	1.5×10^{-1}	2.9×10^{-1}

[*] These authors contributed equally.
[†] These authors also contributed equally.
[‡] Corresponding author: browning@maths.ox.ac.uk or HEnderling@mdanderson.org

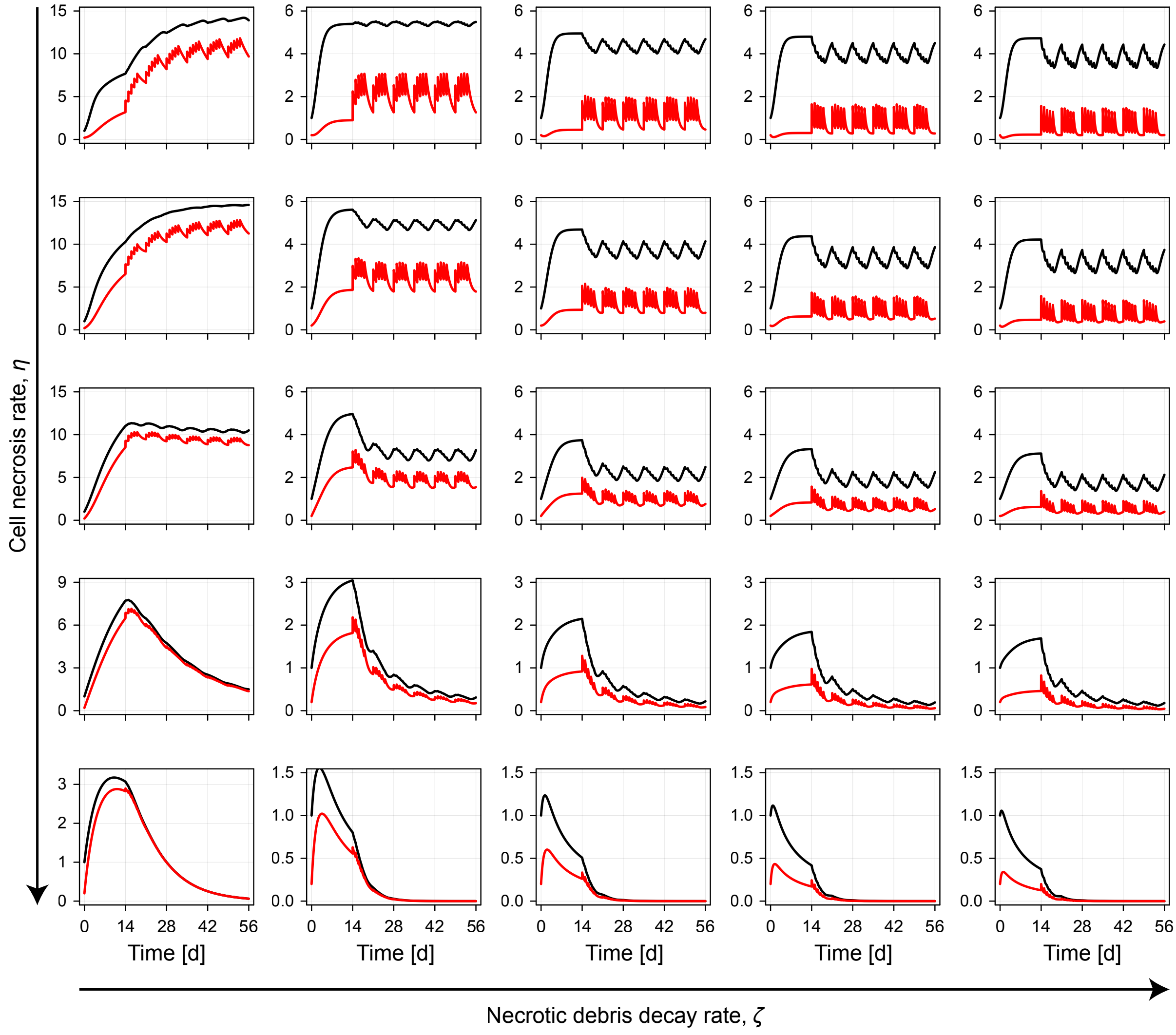

Figure S1. Two parameter sweep of mathematical model. We sweep across the parameters $\zeta \in \{0.1, 0.5, 1, 1.5, 2\}$ and $\eta \in \{0.1, 0.25, 0.5, 0.75, 1\}$ with the other parameters fixed at $\lambda = 1$, $K = 5$, $\gamma = 0.3$, and $\phi_0 = 0.2$. Arrows indicate the direction of increasing ζ and η. Trajectories show the total tumour volume (black), and necrotic volume (red). All patients undergo the standard course of treatment used in the classification procedure in the main document (daily doses of radiotherapy on weekdays over a six week period, initiated from $t = 14\,\mathrm{d}$).

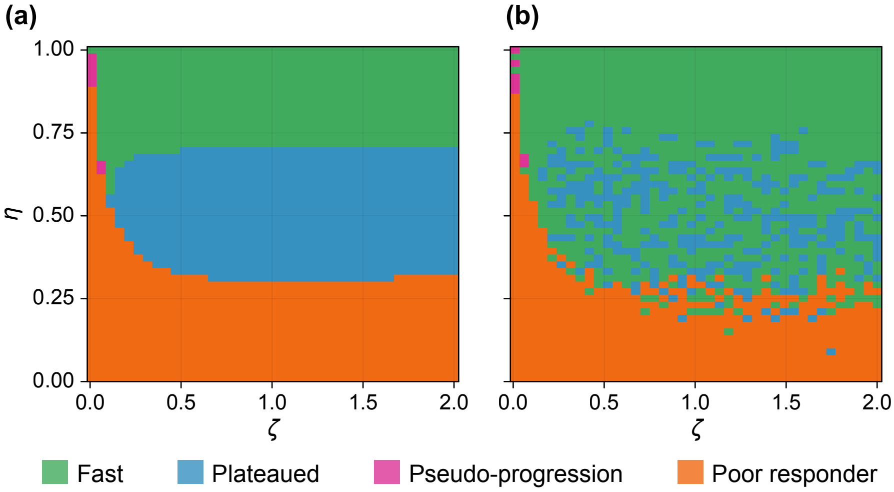

Figure S2. Classified patient responses from two parameter sweep. We sweep across the parameters $0 < \zeta \leq 2$ and $0 < \eta \leq 1$ with the other parameters held fixed at $\lambda = 1$, $K = 5$, $\gamma = 0.3$, and $\phi_0 = 0.2$. All patients undergo the standard course of treatment used in the classification procedure in the main document (daily doses of radiotherapy on weekdays over a six week period, initiated from $t = 14\,\mathrm{d}$). In (a), classification is performed using noise-free synthetic data, whilst in (b), classification is performed using noisy synthetic data produced using the statistical model with pre-estimated noise parameters.

Table S2. $\hat{R}$ MCMC diagnostic statistics for each patient.

ID	In Training	λ	K	γ	ζ	η	ϕ_0
1	Yes	1.003	1.001	1.004	1.005	1.002	1.003
2	Yes	1.001	1.002	1.0	1.0	1.001	1.002
3	Yes	1.025	1.017	1.003	1.007	1.007	1.007
4	Yes	1.0	1.001	1.003	1.001	1.002	1.002
5	No	1.067	1.051	1.063	1.021	1.029	1.047
6	No	1.0	1.003	1.0	1.0	1.0	1.001
7	Yes	1.001	1.003	1.001	1.001	1.001	1.001
8	Yes	1.003	1.004	1.001	1.001	1.001	1.0
9	Yes	1.002	1.001	1.002	1.002	1.001	1.003
10	Yes	1.006	1.004	1.003	1.001	1.008	1.008
11	Yes	1.008	1.002	1.009	1.003	1.008	1.006
12	Yes	1.001	1.0	1.007	1.002	1.002	1.001
13	No	1.011	1.017	1.015	1.002	1.002	1.014
14	No	1.005	1.004	1.004	1.001	1.005	1.002
15	Yes	1.006	1.005	1.007	1.003	1.009	1.002
16	Yes	1.001	1.002	1.001	1.002	1.002	1.003
17	Yes	1.001	1.001	1.001	1.001	1.001	1.003
18	Yes	1.0	1.0	1.0	1.0	1.002	1.0
19	Yes	1.004	1.003	1.009	1.006	1.004	1.001
20	Yes	1.011	1.009	1.009	1.011	1.008	1.025
21	No	1.001	1.002	1.0	1.003	1.001	1.001
22	Yes	1.008	1.003	1.007	1.004	1.012	1.004
23	Yes	1.006	1.0	1.007	1.003	1.009	1.005
24	Yes	1.0	1.001	1.0	0.999	1.001	1.001
25	No	1.001	1.001	1.0	1.001	1.003	1.002
26	Yes	1.002	1.001	1.001	1.003	1.002	1.001
27	Yes	1.001	1.0	1.0	1.0	1.0	1.0
28	Yes	1.003	1.007	1.006	1.015	1.004	1.008
29	No	1.003	1.001	1.0	1.001	1.002	1.001
30	Yes	1.002	1.002	1.003	1.001	0.999	1.0
31	Yes	1.0	1.0	1.001	1.001	1.001	1.0
32	Yes	1.001	1.001	1.0	1.0	1.0	1.001
33	Yes	1.003	1.001	1.0	1.001	0.999	1.002
34	No	1.001	1.002	1.002	1.002	1.0	1.001
35	Yes	1.01	1.001	1.003	1.007	1.009	1.008
36	No	0.999	1.001	1.0	1.001	1.001	1.001
37	Yes	1.004	1.004	1.001	1.002	1.003	1.004
38	Yes	1.0	1.001	1.006	1.001	1.003	1.001
39	Yes	1.004	1.004	1.003	1.0	1.003	1.002
40	Yes	1.013	1.004	1.01	1.001	1.004	1.008
41	No	1.004	1.005	1.009	1.009	1.003	1.003
42	Yes	1.002	1.0	1.001	1.001	1.001	1.001
43	Yes	1.003	1.003	1.001	1.002	1.0	1.003
44	Yes	1.0	1.001	1.0	1.001	1.001	1.0
45	Yes	1.134	1.208	1.1	1.121	1.129	1.296
46	Yes	1.001	1.0	1.0	1.002	1.003	1.001
47	Yes	1.002	1.004	1.008	1.001	1.009	1.001
48	Yes	1.43	1.575	1.447	1.781	1.715	1.537
49	Yes	1.001	1.0	1.0	1.001	1.0	0.999
50	Yes	1.003	1.002	1.008	1.001	1.008	1.004
51	No	1.002	1.04	1.001	1.003	1.0	1.0

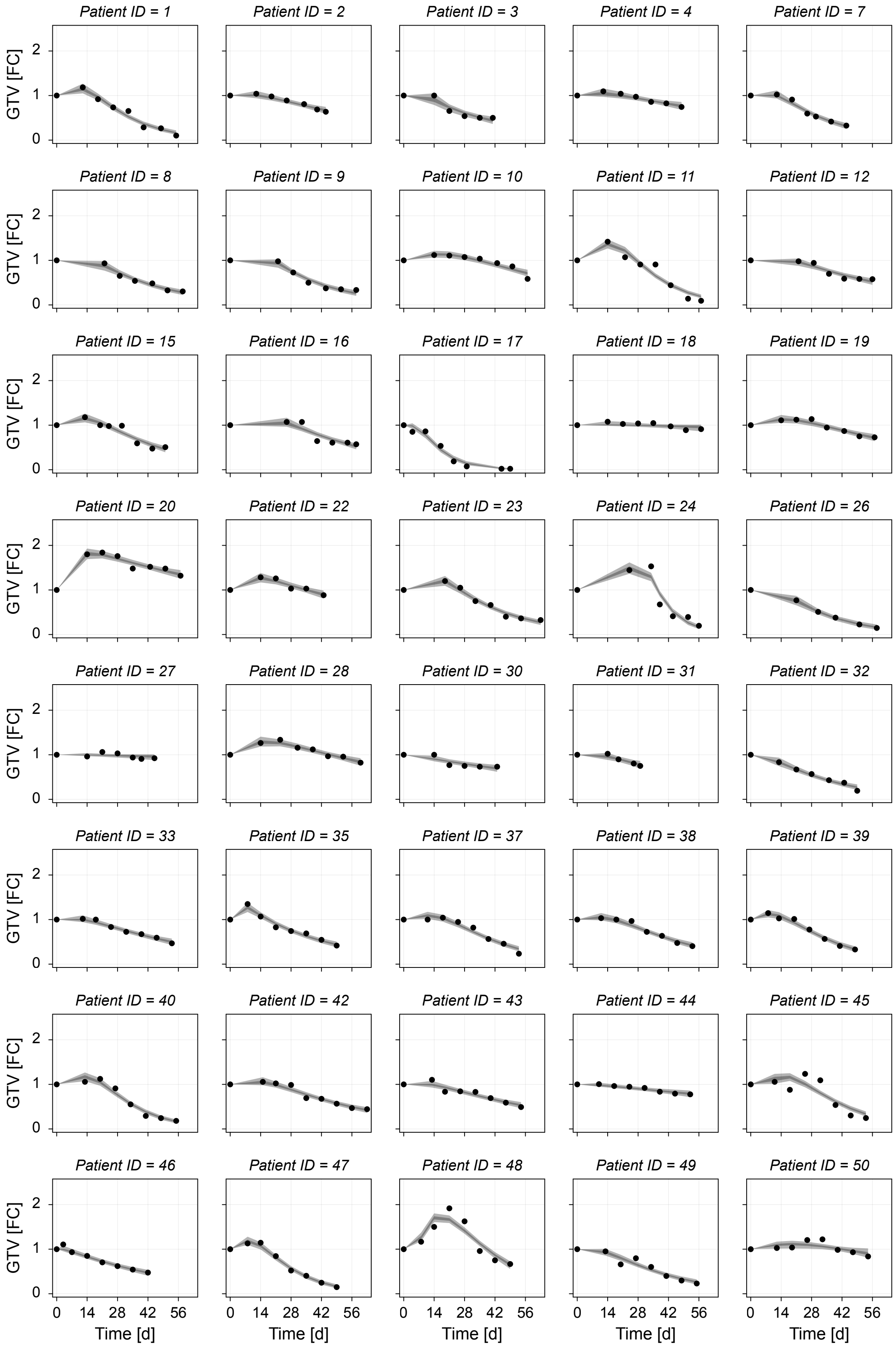

Figure S3. Fits for patients in the training set. Individual fits for all patients in the training set, using the population-level prior. Shown are the data (black disc), 50% credible interval (light grey), and 95% credible interval (dark grey). Note that model predictions are only drawn at times corresponding to clinical measurements: results for intermediate time points show as a linear interpolation.

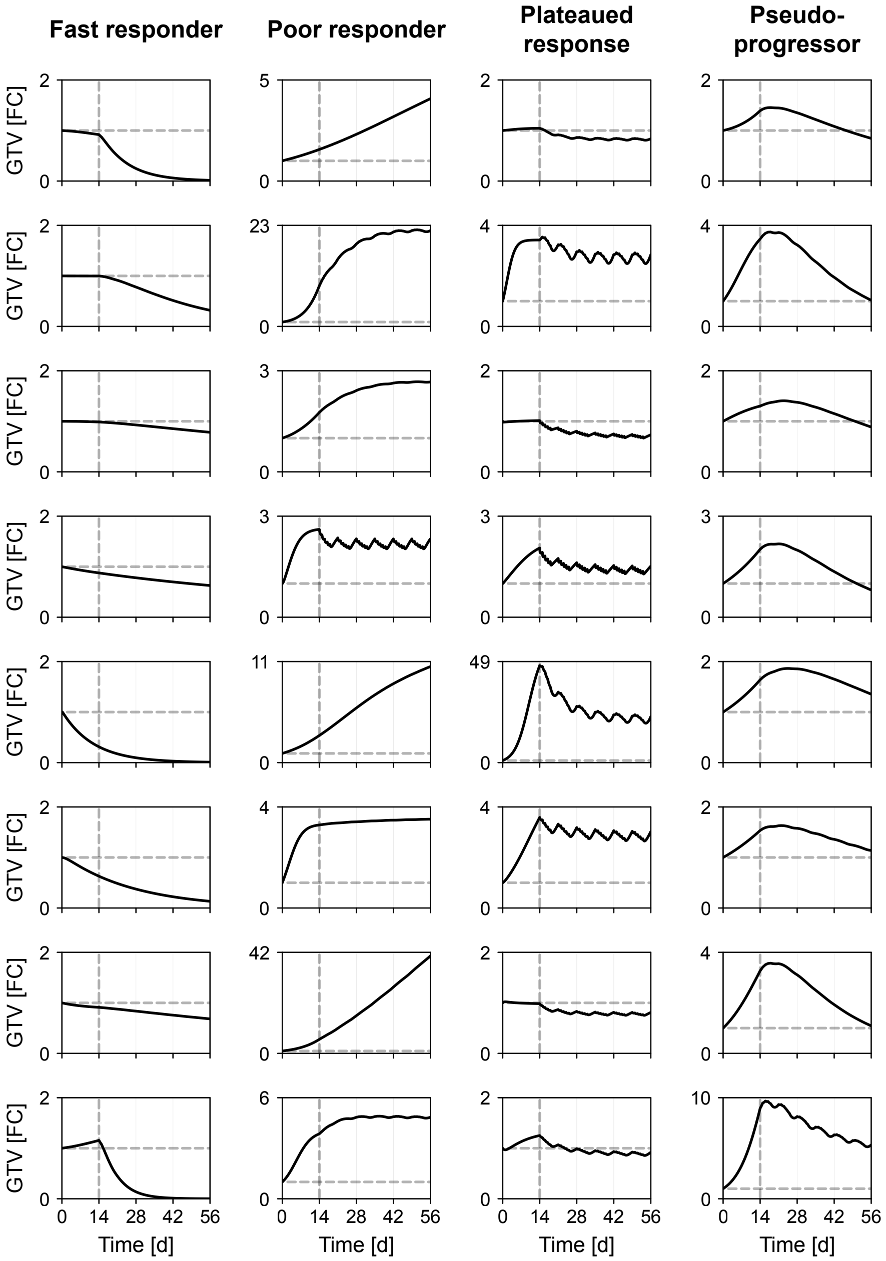

Figure S4. Subset of classified prior samples. We demonstrate the choices in the classification algorithm by simulating eight patients of each class from the prior distribution. All patients undergo the standard course of treatment. Shown is the tumour volume (black), a horizontal line indicating unity (dashed grey), and a vertical line showing the time of the first radiotherapy dose (dashed grey).

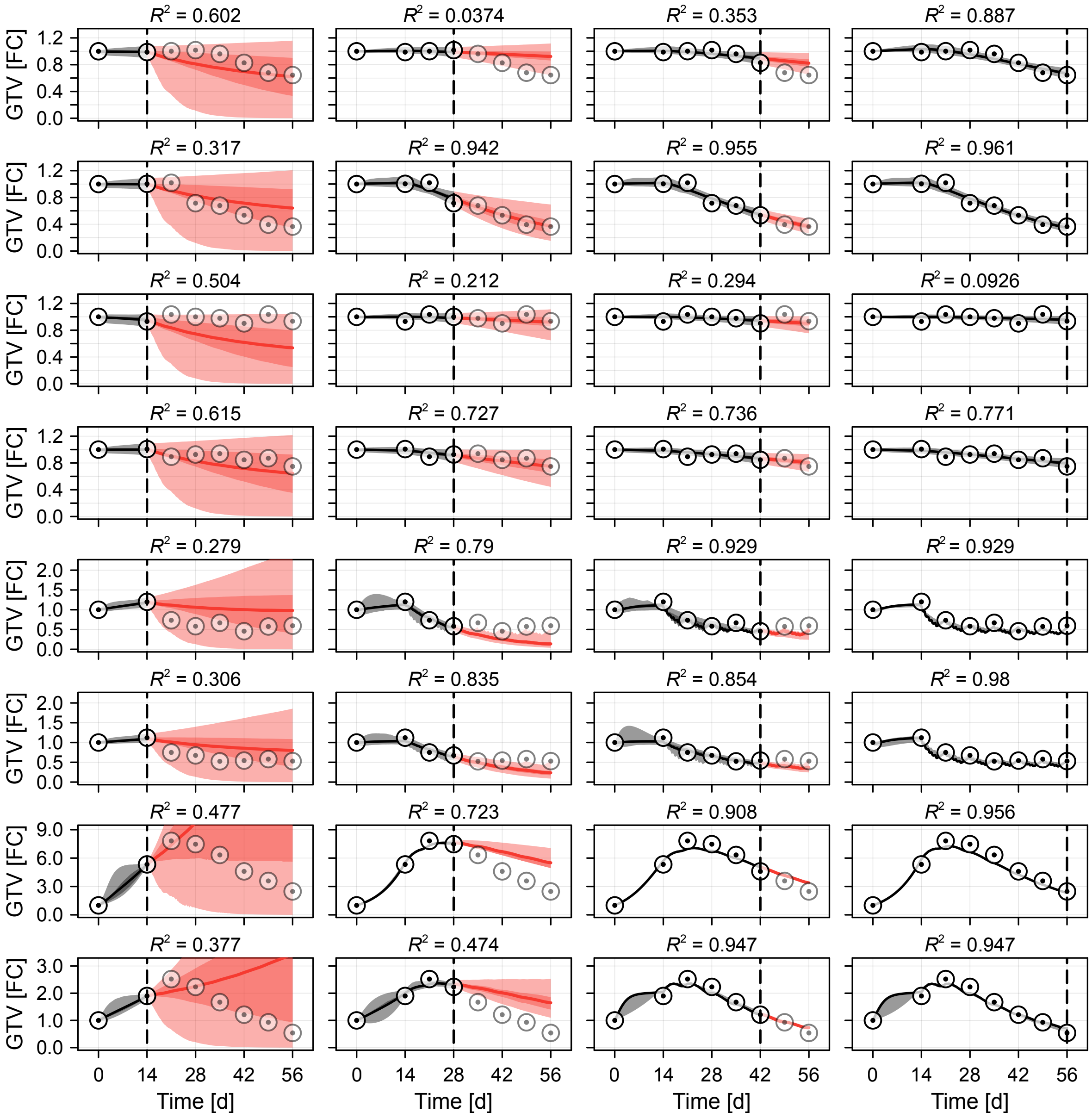

Figure S5. Results for eight additional synthetic patients. We reproduce the analysis from Fig. 5 in the main document for eight additional synthetic patients (two from each response classification).

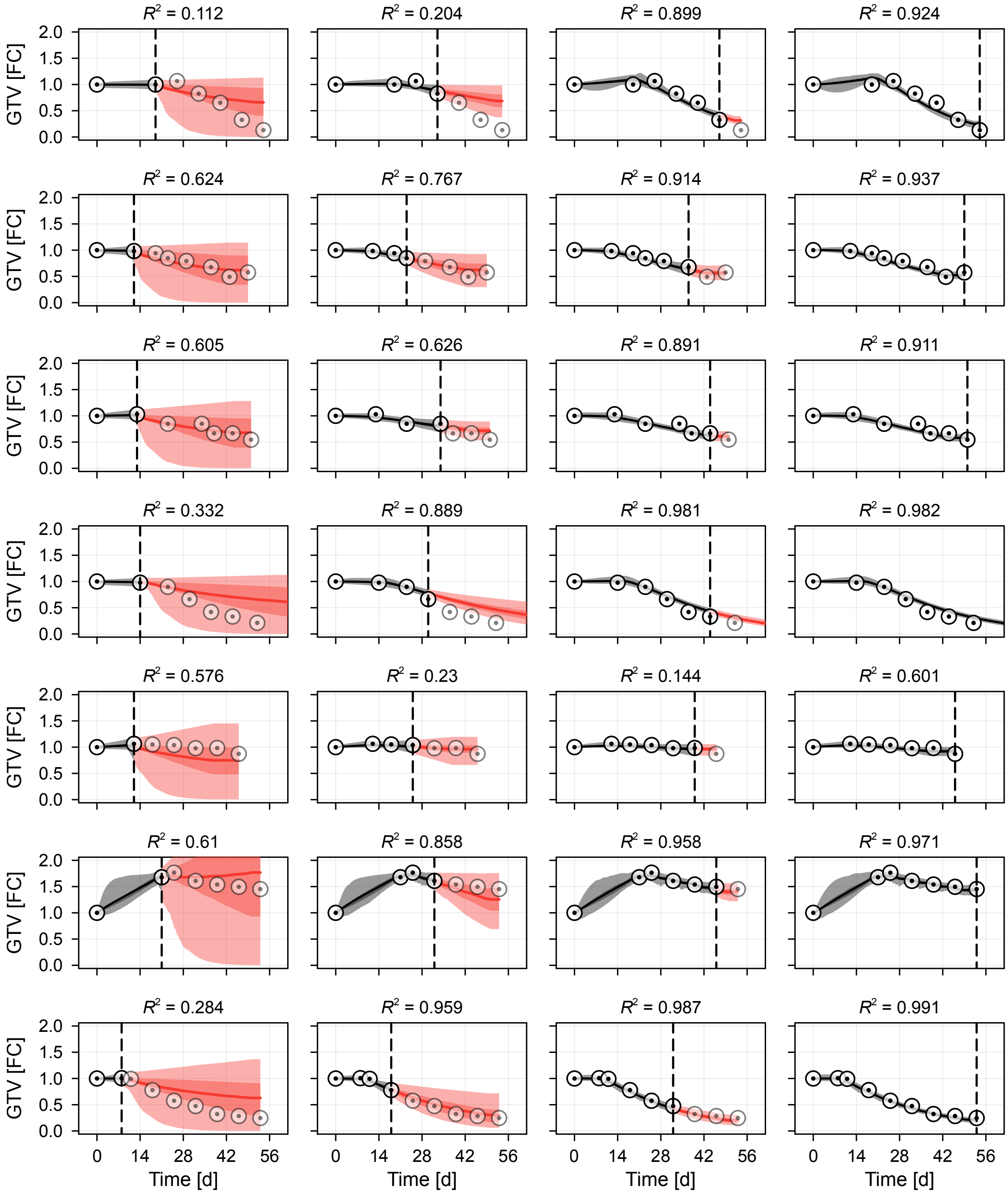

Figure S6. Temporal predictions for the seven patients excluded from the training set that do not appear in Fig. 7 of the main document. We reproduce the analysis from Fig. 5 and Fig. 7 in the main document for those remaining patients that were excluded from the training set. Each row corresponds to a single patient. These patients were not included in the training set, and so their results are representative of clinical predictions made throughout a new patient's course of treatment.

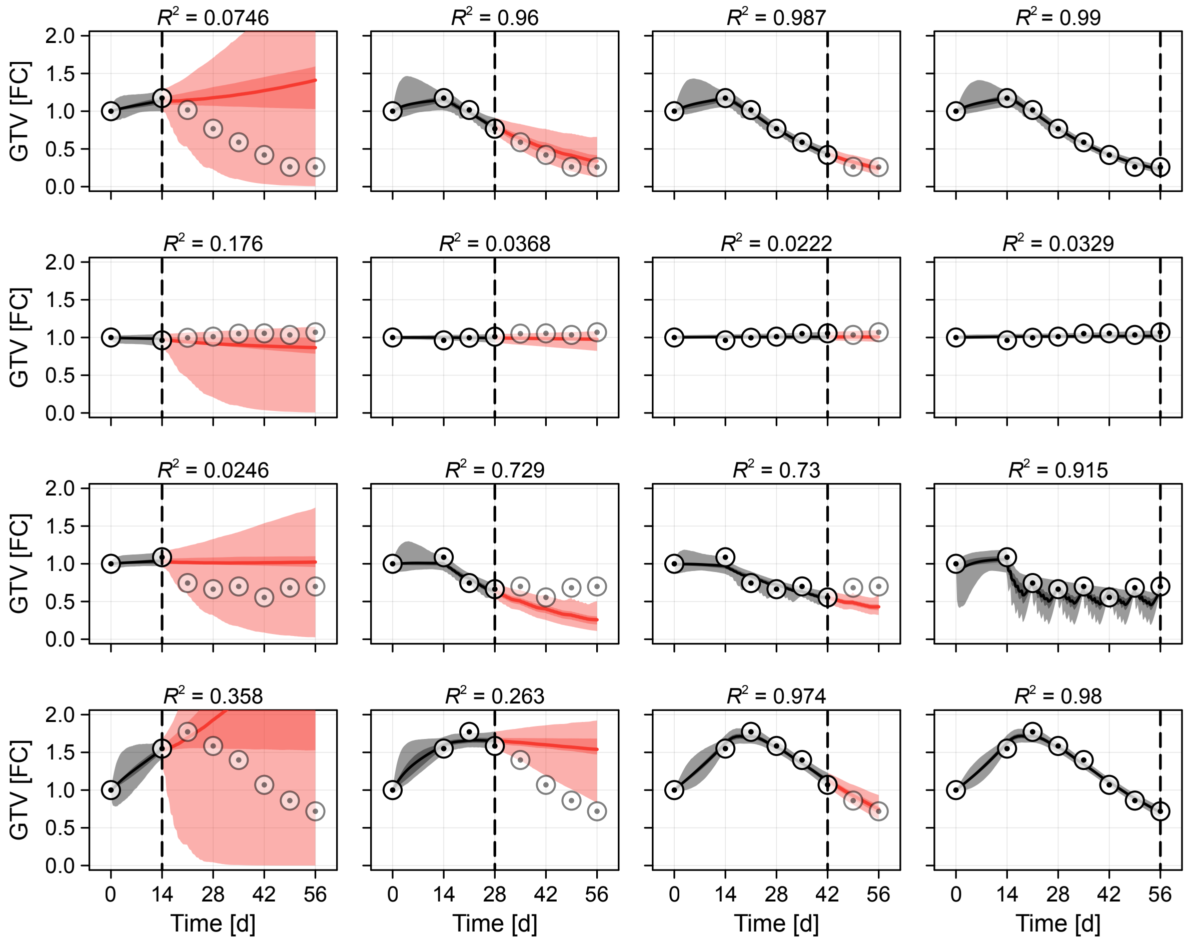

Figure S7. Temporal predictions from four synthetic patients using the uninformative prior. We reproduce the analysis from Fig. 5 in the main document using the uninformative (i.e., population-level) prior.

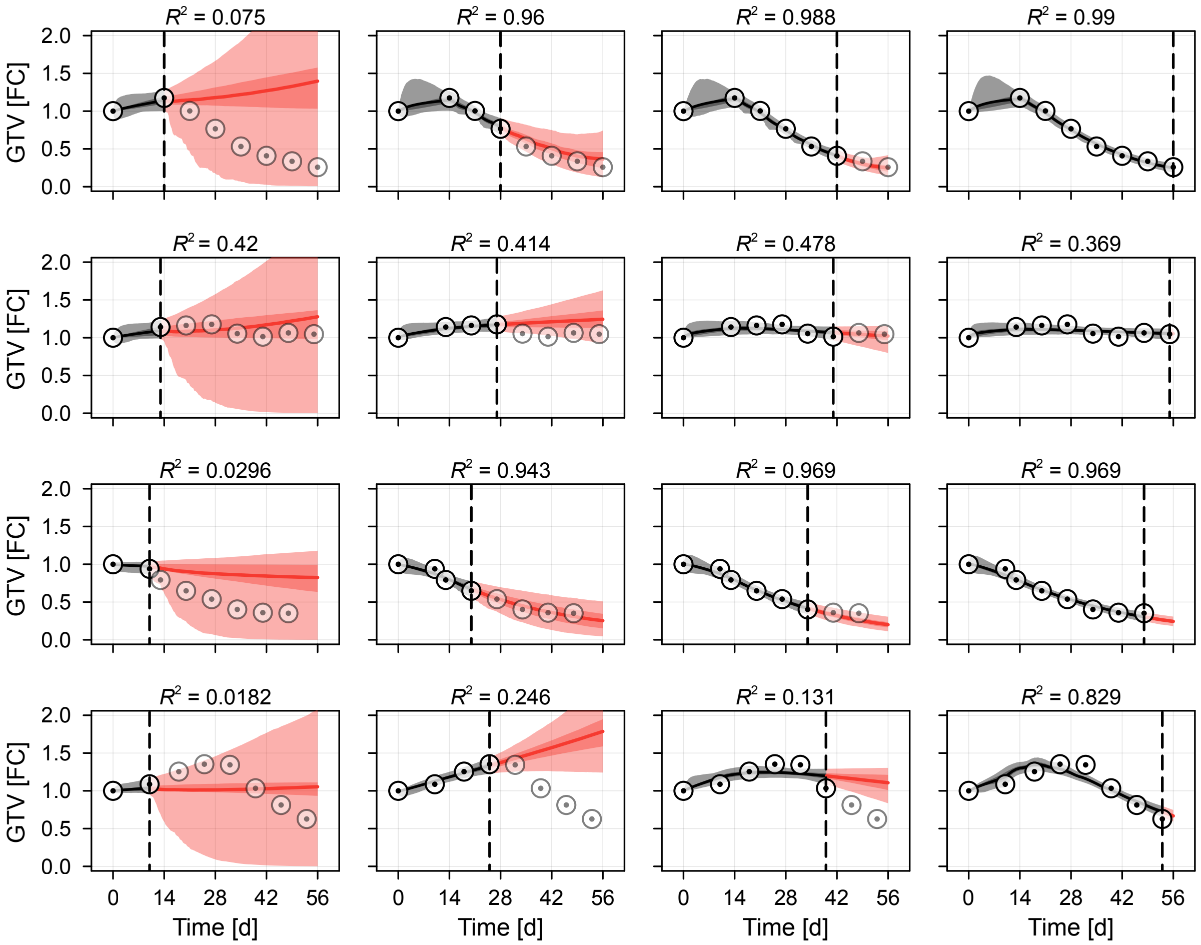

Figure S8. Temporal predictions from four patients excluded from the training set using the uninformative prior. We reproduce the analysis from Fig. 7 in the main document using the uninformative (i.e., population-level) prior.

Comparison to Bayesian hierarchical approach

Here, we compare predicted uninformed patient trajectories from the pseudo-hierarchical approach presented in the main document, to a standard Bayesian hierarchical model implemented in `Turing.jl` [1].

We assume that, at the population-level, the logarithm of each model parameter is distributed according to a truncated normal distribution, with support given by the support of the uniformed prior as implemented in the main text. For example,

$$\log \lambda \sim \text{TruncatedNormal}(\mu_\lambda, \sigma_\lambda, \lambda_{\text{min}}, \lambda_{\text{max}}), \tag{1}$$

where the prior for λ in the main text was given by $\log \lambda \sim \text{Uniform}(\lambda_{\text{min}}, \lambda_{\text{max}})$. The population-level priors are given by

$$\mu_\lambda \sim \text{Uniform}(\lambda_{\text{min}}, \lambda_{\text{max}}), \tag{2a}$$

$$\log \sigma_\lambda \sim \text{Uniform}(-10.0, 3.0), \tag{2b}$$

where the prior for the log standard deviation is chosen to span a sufficiently wide range of scales that model parameters may either be concentrated, or approximatately uniformly distributioned (i.e., a truncated normal distribution with untruncated standard deviation much larger than the support of the truncated distribution). The priors for the other parameters K, γ, ζ, η, and ϕ_0 are given in the same way. The noise parameters, α_1 and α_2, are inferred simultaneously.

Since the individual dosing regime of each patient effectively prescribes a different mathematical model for each patient, the hierarchical problem is potentially much more computationally costly than the pseudo-hierarchical approach. As such, we demonstrate the hierarchical approach on a randomly chosen subset of the training data, comprising $\tilde{N} = 10$ patients. We perform inference using `Turing.jl`'s inbuilt Hamiltonian Monte Carlo algorithm (code available on GitHub*), and sample four independent chains, each of 50,000 samples.

Results in fig. S9 highlight the fundamental differences between a standard hierarchical approach and the resampling-based approach presented in the main text. In particular, it is not straightforward to infer a correlation structure between the six patient-level parameters in a standard hierarchical approach, and so we do not gain information about parameter correlations or multimodality arising from the different patient responses (fig. S9a,b). This is problematic for prediction, since the correlation structure and multimodality (recovered using the pseudo-hierarchical approach) constrains model predictions to the space of trajectories seen in the training data. We demonstrate this in3 fig. S9c,d. Predictions from new patients (drawn before any patient-level data is observed) are often unrealistic, and are clearly not constrained to the set of observations drawn from the training data.

*`https://github.com/ap-browning/clinical_predictions/blob/main/figures/figS9.jl`

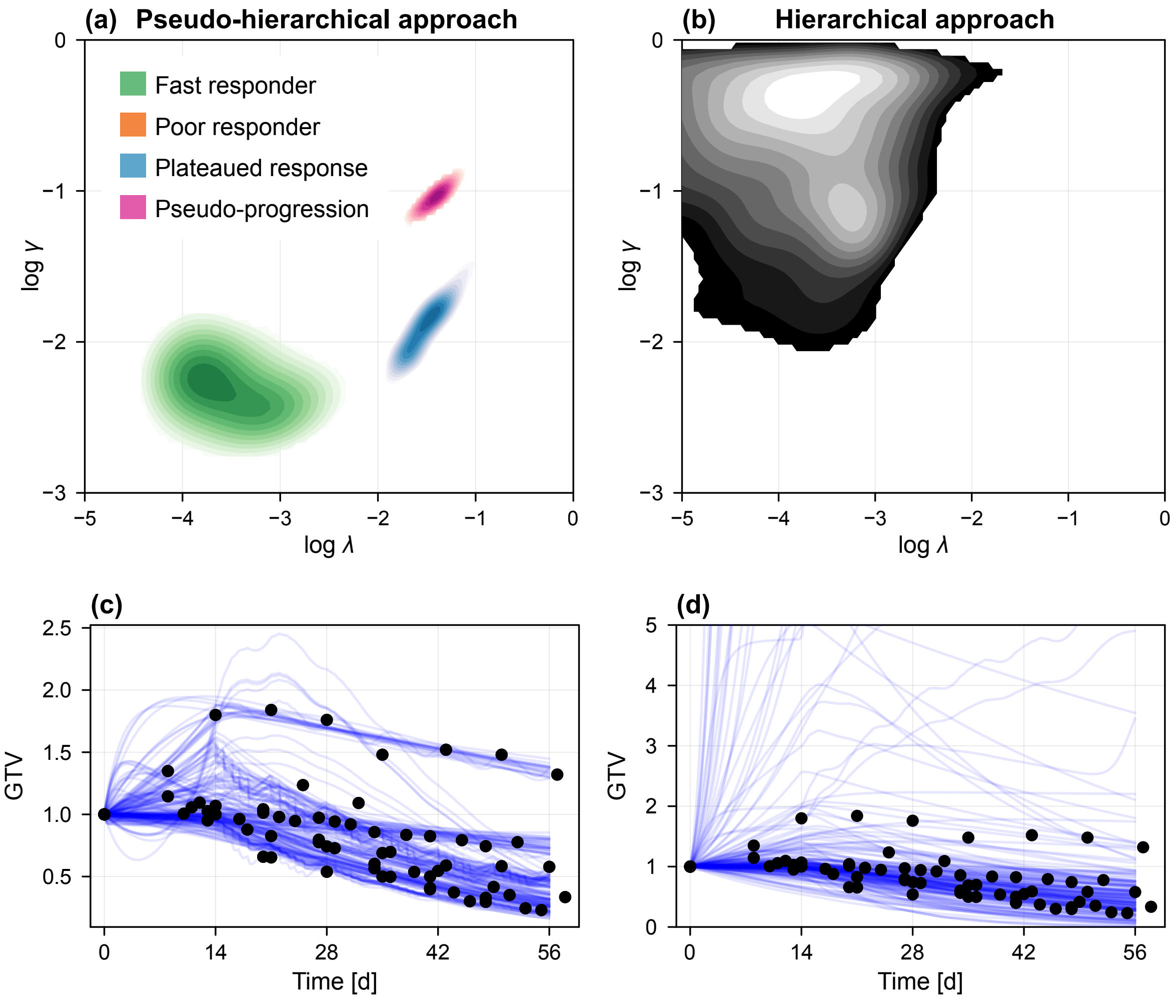

Figure S9. Comparison between the pseudo-hierarchical method and a standard Bayesian hierarchical method. (a,b) Bivariate posterior distribution for patient-level parameters $\log \lambda$ and $\log \gamma$. In (a), parameter distributions are drawn from the mixture of individual posterior distributions for the 10 subjects in the resampled training set. In (b), the distribution is constructed by resampling from the posterior distributions for μ_λ and $\log \sigma_\lambda$, at each sample reconstructing and then sampling from the distribution for $\log \lambda$ given by eq. (1) (and similar for parameters related to γ). (c,d) Predictions drawn from the patient-level distributions formed using (c) the pseudo-hierarchical approach, and (d) the hierarchical approach, for patients undergoing the standard course of treatment used to classify patients (see Section 2.2.1 of the main document). Also shown are GTV measurements from the $\tilde{N} = 10$ subjects in the resampled training set.

References

[1] Ge H, Xu K, Ghahramani Z. 2018 Turing: a language for flexible probabilistic inference. In *International Conference on Artificial Intelligence and Statistics, AISTATS 2018, 9-11 April 2018, Playa Blanca, Lanzarote, Canary Islands, Spain* pp. 1682–1690.
